# A computational account of threat-related attentional bias

**DOI:** 10.1371/journal.pcbi.1007341

**Published:** 2019-10-10

**Authors:** Toby Wise, Jochen Michely, Peter Dayan, Raymond J. Dolan

**Affiliations:** 1 Wellcome Centre for Human Neuroimaging, University College London, London, United Kingdom; 2 Max Planck UCL Centre for Computational Psychiatry and Ageing Research, University College London, London, United Kingdom; 3 Max Planck Institute for Biological Cybernetics, Tübingen, Germany; UNITED KINGDOM

## Abstract

Visual selective attention acts as a filter on perceptual information, facilitating learning and inference about important events in an agent’s environment. A role for visual attention in reward-based decisions has previously been demonstrated, but it remains unclear how visual attention is recruited during aversive learning, particularly when learning about multiple stimuli concurrently. This question is of particular importance in psychopathology, where enhanced attention to threat is a putative feature of pathological anxiety. Using an aversive reversal learning task that required subjects to learn, and exploit, predictions about multiple stimuli, we show that the allocation of visual attention is influenced significantly by aversive value but not by uncertainty. Moreover, this relationship is bidirectional in that attention biases value updates for attended stimuli, resulting in heightened value estimates. Our findings have implications for understanding biased attention in psychopathology and support a role for learning in the expression of threat-related attentional biases in anxiety.

## Author summary

To make inferences and learn efficiently in the face of a multiplicity of stimuli we need to allocate attention preferentially to those that are most motivationally relevant. It is unclear how this is achieved in aversive environments. We investigated how value (the likelihood of an unpleasant event) and uncertainty (akin to ignorance about its probability) influence visual attention during aversive learning. Our results show that attention is influenced by value but not by uncertainty. Attention in turn results in heightened value estimates for attended stimuli. The findings have implications for understanding the development of pathological threat-related attentional biases that are a feature of anxiety disorders.

## Introduction

To enable efficient learning and inference about the environment, perceptual inputs need to be prioritised appropriately. Attention acts as a perceptual filter in sensory processing [1,2], learning [3–5], and inference [6,7], with various statistical [8,9] and computational [10,11] treatments indicating how it might optimally, or approximately optimally, operate. In the face of limited perceptual and cognitive resources, and external noise, an agent needs to select which stimuli need to be attended, and which can be disregarded.

Theoretical work suggests that the factors guiding attention should be task-dependent, with a particular distinction between learning and prediction [9]. When making predictions, and depending on the loss function, attention should generally favour less uncertain predictors [4]. By contrast, when learning, attention should often be directed to those stimuli about which we are most uncertain, and thus have the most to learn [3,9,12]. Importantly these models focus on attentional allocation during competitive learning and prediction, in which an agent needs to act upon a single stimulus selected from multiple rivals [3,4]. In forming an accurate representation of the environment, we frequently face situations requiring multitasking, where we must concurrently maintain and update value estimates for multiple stimuli.

Research on value-guided perceptual attention has focused largely on tasks requiring choice between competing stimuli associated with predetermined levels of reward, reflecting either their innate value or that acquired following learning. Here, studies suggest the value of options influences an attentional priority map, determining a focus for visual selective attention [13–16]. By contrast, other studies provide evidence that visual attention is value-independent [7].

The allocation of attention can also influence choice, for instance by biasing evidence accumulation regarding the value of stimuli [6,7,17]. However, despite a rich literature detailing how receipt of outcomes impact an agent’s value estimates [12,18–23], it remains unclear how value-based *learning* guides perceptual attention at the point of value updating, and vice versa, particularly when we need to learn about multiple stimuli concurrently. Crucially, the majority of tasks investigating attention during value learning focus on choice, following value updating, rather than focusing on the point at which a value update occurs. A number of studies have examined attention allocation at the point of updating during value-free perceptual associative learning [24–27]. However, to our knowledge only one previous study has investigated perceptual attention in a value learning task, showing a bidirectional relationship between value estimates and attention [28]. This suggests visual selective attention during competitive learning is guided by factors relevant to the learning process, while learning is itself guided by attention in a manner reminiscent of the effects of attention on choice behaviour [7,29].

The above issues are critical in motivationally aversive or threatening environments, given the importance of detecting and avoiding potential threats relative to harvesting modest gains. The aversive case is of particular interest due to an additional influence from Pavlovian behavioural biases differently related to learning. Thus, threatening stimuli engender reflexive avoidance behaviour [30–32], raising a theoretical possibility that visual attention is subject to Pavlovian repulsive effects. Conversely, both rodents and humans display “risk assessment” behaviour when faced with threat [32–35], which may result in an increased allocation of visual attention [36–38]. While the influence of aversive value and irreducible uncertainty on attention have been studied, typically showing effects of value [36,39], this work has not examined how attention functions at the point of value updating, and has not investigated the effect of reducible uncertainty. Importantly, the bidirectional interaction between learning and attention has not been studied in the aversive domain despite its relevance to psychopathology. Anxious individuals show biased attention towards potentially threatening stimuli [40,41], and this is thought to be a causal factor in the development and maintenance of an anxious state [42]. Likewise there is evidence for impaired learning processes in clinical anxiety [43–46]. As it is unclear whether aversive learning is influenced by visual attention, as is the case in the reward domain [28], resolving this issue could provide a potential explanation for reported effects of attention towards threat on the experience of subjective anxiety [42].

In this study, we examine how learned value and uncertainty guide visual selective attention while subjects engage in concurrent aversive learning (and unlearning) about multiple, rapidly changing, stimulus values. While an established literature has demonstrated that amygdala-dependent fear memories persist over extended timescales [47,48], behavioural findings indicate that human subjects can track rapid changes in contingencies [43,49], while animal findings suggest that such rapid learning and unlearning may be mediated by brain structures other than amygdala with its well known involvement in more persistent forms of fear memory [50].

We designed a task in which value and uncertainty were independently manipulated. Subjects were required to learn the aversive value of multiple visual stimuli (operationalised as the likelihood of receiving mild electric shocks) concurrently, while visual attention was monitored using eye tracking. Importantly, we investigated how limited perceptual attentional resources are allocated when subjects concurrently update value estimates about multiple stimuli. This addresses a different question to an existing literature that has focused on the role of attention in assigning a single outcome to one of multiple competing stimuli that could have generated this outcome. Here, attention is prioritised based on a need to allocate limited resources appropriately during a relatively brief value updating period, but may also be engaged by known biases in attention towards threatening stimuli [41]. Specifically, we tested whether preparatory visual attention prior to outcome receipt is guided by aversive value (i.e. the probability of a negative outcome) and reducible uncertainty (i.e. the variance around this estimate), based on subject-specific estimates of these quantities informed by a computational model of task behaviour. In follow-up analyses, we then assessed whether this relationship was bidirectional, asking whether attention during outcome receipt influenced subsequent value judgements. Our findings reveal that aversive value, but not uncertainty, guides visual selective attention in a bidirectional manner, with subjects updating value estimates to a greater extent when a stimulus was the object of attention.

## Materials and methods

### Ethics statement

The study was approved by the UCL Research Ethics Committee (reference 9787/001). All subjects provided written consent.

### Preregistration

The primary hypotheses and methods (including the model space and measures of visual attention) for this study regarding effects of learning on attention were preregistered on the open science framework (https://osf.io/8rwcu/register/5771ca429ad5a1020de2872e). Non-preregistered analyses, including those investigating effects of attention on learning, are treated as exploratory. All exclusion criteria were determined through piloting and were included in the preregistration.

### Participants

We recruited 65 participants (40 female, mean (SD) age = 26.67 (8.93)) from subject databases at University College London. All participants provided informed consent and were compensated for participation. Prior to analysis, two subjects were excluded due to not providing full behavioural data.

### Aversive learning task

Participants completed an aversive learning task featuring two stimuli that were each independently associated with varying probabilities of electric shocks. Shock likelihood was biased towards 0% (mean shock probability across all trials = 36%) to ensure subjects were exposed to a tolerable number of shocks, but the exact probability fluctuated across the task such that one stimulus had a stable probability while the other varied. This manipulation was intended to ensure that both the actual shock probability, and the uncertainty around this probability, varied over the course of the task. Subjects were fully informed about this aspect of the task and were instructed to keep track of these variations in order to achieve accuracy. We did not incentivise accuracy with money so as to prevent any possible reward-related learning processes. The experimental setup meant subjects could receive shock from any combination of stimuli.

Shock intensity was calibrated using a titration procedure to ensure shocks had an equivalent subjective impact across subjects [51,52]. In brief, subjects were exposed to a series of shocks that gradually increased in strength and were asked to rate unpleasantness of the shock on a scale from 1 to 10, where 10 indicated the maximum they would be willing to tolerate. This procedure was repeated three times, and 80% of the average 10-rated current level was used for the experiment.

**Fig 1:**
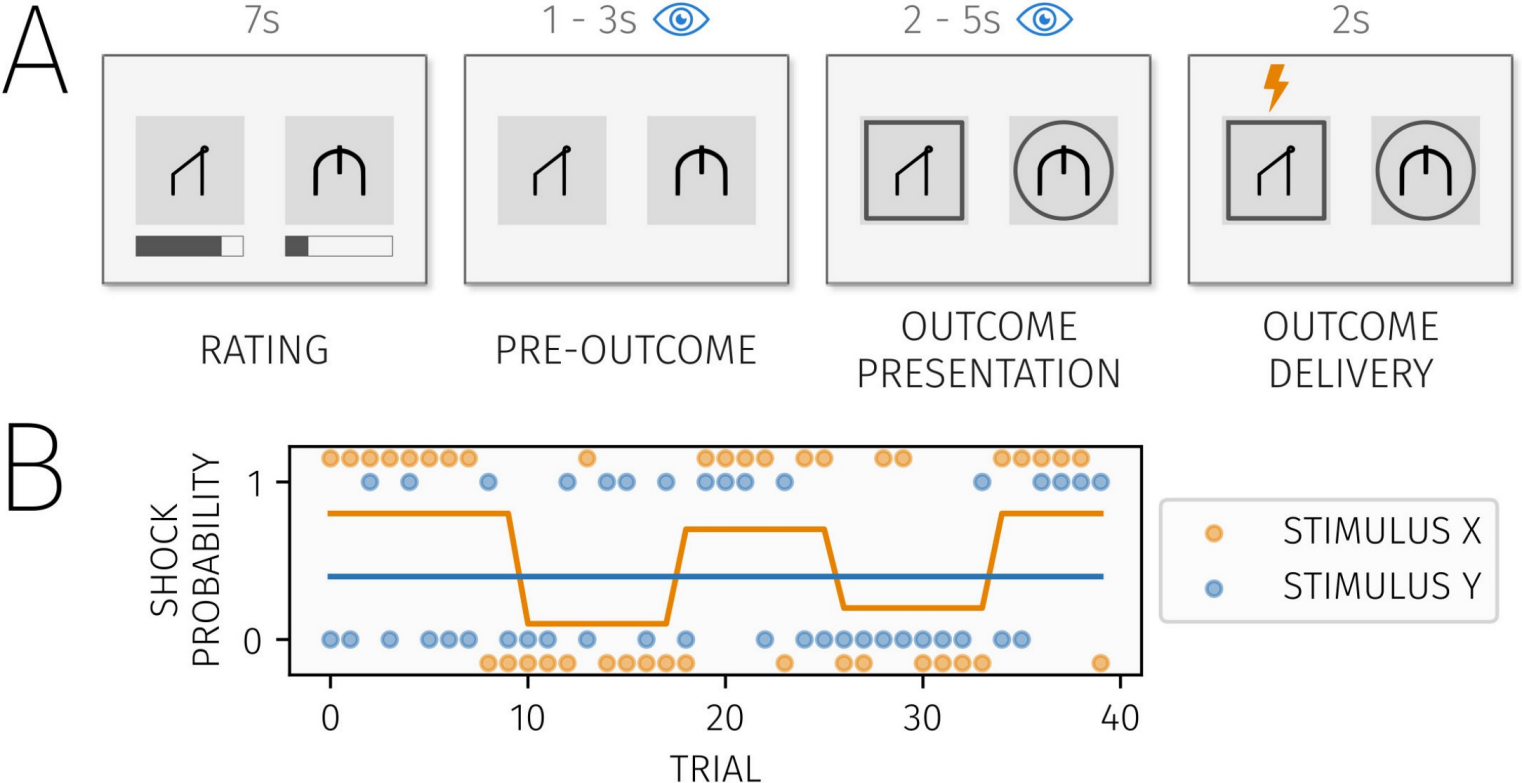
A) Trial sequence. On each trial, two stimuli were presented simultaneously during a "rating" period. Subjects estimated the probability of an upcoming shock for each stimulus using rating bars displayed either below or above the stimuli. When subjects indicated their response the rating bars disappeared. The stimuli remained on the screen for 1–3 seconds in a “pre-outcome” period. Next, outcomes for both stimuli were presented concurrently in an “outcome presentation” period. Here, a square frame around a stimulus indicated an upcoming shock while a circle indicated no shock. Finally, either two, one or no shock was administered during the “outcome delivery” period. For analyses of learning effects on attention, and attention effects on learning, we used eye tracking data where the focus was on pre-outcome and outcome phases respectively. B) Shock probability and an exemplar outcome sequence for one of the four task blocks. The blue and orange lines represent the generative shock probability level for each of the two stimuli, while the circles represent the outcomes on each trial (1 representing a shock and 0 representing no shock).

The two task stimuli were presented simultaneously on screen over four trial phases (Fig 1). In a rating phase, subjects indicated the likelihood with which each stimulus predicted a shock at the current moment in time, i.e., the expected probability (value) of each stimulus. Subjects provided ratings by moving rating bars shown either above or below the stimuli. Subjects were given 7 seconds to provide these ratings, and instructed to provide these as fast and accurately as possible. If they did not believe the probability had changed since their last rating, they could opt to leave the bar in the position they set on the previous trial.

After a brief fixation cross, subjects were again shown the stimuli in the same position as the rating phase for 1–2 seconds (randomly jittered on each trial). We refer to this as the pre-outcome phase, corresponding to the period of our eye-tracking fixation analysis. After a further brief delay, the outcome for each stimulus was presented simultaneously for 2-5s. Here, an upcoming shock was indicated by a square over the stimulus, while a no-shock outcome was indicated by a circle. Subsequently, outcomes were delivered: If both stimuli indicated a shock, they were presented one after the other in a random order, with a shock icon shown concurrently over the delivering image to render the stimulus-outcome association clear. This served to separate learning about the outcome from the delivery of outcomes. After a fixed inter-trial interval of 2s, the next trial started with presentation again of stimuli and rating bars. On each trial, the side on which an individual stimulus appeared was randomly determined to prevent any bias towards to one or other side of the screen.

Subjects completed four blocks of 40 trials, with a short break between blocks. The exact outcome probability sequence was different in each block, and the order of blocks was randomly determined for each subject. Subjects were informed that shock probabilities would change at the start of each new block, and they should disregard anything they had learned on the previous block. The allocation of visual stimuli to each probability sequence was counterbalanced across subjects. A different pair of stimuli was used in each block, with two stimuli being selected at random from a pool of four potential stimuli. This was designed to allow variation in stimuli across blocks, while limiting effects of perceptual novelty.

### Computational modelling of behaviour

We used computational modelling of behaviour to capture the processes governing learning and to generate uncertainty measures for further analysis. We tested five computational models comprising three reinforcement learning (RL) models and two probabilistic models. The first of the RL models (Model 1, Eq 1) was a basic Rescorla-Wagner model where value (represented by estimated shock probability, *V*) is updated every trial (represented by *t*) according to a prediction error (PE) weighted by a free learning rate free parameter *α*.

$${\text{V}}_{\text{t}+1}^{\text{X}}={\text{V}}_{\text{t}}^{\text{X}}+\alpha \;\cdot \left({\text{outcome}}_{\text{t}}^{\text{X}}-{\text{V}}_{\text{t}}^{\text{X}}\right)$$ (1)

Here, V^X^ refers to the value of the one of the stimuli (presented on either the left or right of the screen depending on the trial), while outcome^X^ refers to the outcome associated with that stimulus on the present trial. Although subjects were informed that shock probabilities for the stimuli were independent, it is possible they generalised the outcome of one stimulus when updating the value of the other stimulus. We accounted for this possibility by adding a second learning signal based on the difference between the estimated value of the current stimulus and the outcome of the other stimulus (outcome^Y^), weighted by a second learning rate *ω* (Eq 2).

$${\text{V}}_{\text{t}+1}^{\text{X}}={\text{V}}_{\text{t}}^{\text{X}}+\omega \cdot \left({\text{outcome}}_{\text{t}}^{\text{Y}}-{\text{V}}_{\text{t}}^{\text{X}}\right)$$ (2)

We also tested a modified version of this first model that had two learning rates for the current stimulus [53]; one for better than expected outcomes (indicated by a positive prediction error) and one for worse than expected outcomes (indicated by negative prediction errors), where both were free parameters (Model 2, Eq 3).

$$\delta_{t}={\text{outcome}}_{\text{t}}^{\text{X}}-{\text{V}}_{\text{t}}^{\text{X}}$$

$${\text{V}}_{\text{t}+1}^{\text{X}}={\text{V}}_{\text{t}}^{\text{X}}+\begin{array}{c} \alpha^{+}\cdot {\delta \;\text{if}\;\delta }_{t}>0\\ \alpha^{-}\cdot {\delta \;\text{if}\;\delta }_{t}<0 \end{array}$$ (3)

We tested a Pearce-Hall/Rescorla-Wagner hybrid model incorporating a dynamic learning rate that depended on the magnitude of recent prediction errors (Model 3, Eq 4), where a larger prediction error on the most recent trial leads to an increased learning rate on the current trial. This results in a learning rate that is highest when an agent receives an indication their current value estimate is incorrect, and should increase learning rate about the current state of the environment. As a result, learning should be highest when shock probabilities change, and lowest when they are stable. The rate at which the learning rate changes is governed by an additional free parameter *k*.

$$\alpha_{\text{t}+1}^{\text{X}}=\alpha_{\text{t}}^{\text{X}}+\text{k}\cdot \left(\delta_{t}^{2}-\alpha_{\text{t}}^{\text{X}}\right)$$ (4)

We also included a second variant of this hybrid Rescorla-Wagner / Pearce-Hall model following Tzovara et al. (54), which updates the learning rate on each trial similarly to the previous model but using the absolute prediction error rather than the squared prediction error (Model 4, Eq 5):

$$\alpha_{\text{t}+1}^{\text{X}}=\alpha_{\text{t}}^{\text{X}}+\text{k}\cdot \left(\left|\delta_{t}\right|-\alpha_{\text{t}}^{\text{X}}\right)$$ (5)

The first probabilistic model we tested was a leaky beta model (Model 5, Eq 6). This is a probabilistic learning model that naturally represents both the shock probability estimate (the mean of a beta distribution) and the uncertainty around this estimate (the variance of the beta distribution). This family of models has been successfully used in modelling reward learning tasks [54], and similar models have been shown to describe behaviour in aversive learning tasks better than reinforcement learning models [55], making it an appropriate candidate model family for the current task. Our model assumes subjects estimate the A and B parameters of a beta distribution over the value of each stimulus, and update these on each outcome at a rate dependent on parameter *τ*. Here, A represents evidence for shock outcomes, while B represents evidence for no-shock outcomes, such that A is updated following a shock outcome while B is updated following a no-shock outcome. This results in a beta distribution that is biased towards the most frequently occurring outcome. Hence, frequently occurring shocks will lead to high values of A and low values of B and a mean of the distribution that is biased towards 1, representing a high shock probability. The “leak” in the model is represented by *λ*, which ensures that estimates are weighted towards more recent outcomes by reducing the accumulating evidence for both outcomes on each trial so that the current trial has a greater impact upon value estimates. This was desirable, since subjects were informed that shock probabilities could change during the task. As in the previously described reinforcement learning models, ω here represents a parameter governing the influence of the other stimulus shown on screen. Although its implementation in these models is different to the previous models, its effect on value estimates is the same.

$${\text{A}}_{\text{t}+1}^{\text{X}}=\left(1-\lambda \right)\cdot {\text{A}}_{\text{t}}^{\text{X}}+\tau \cdot {\text{outcome}}_{\text{t}}^{\text{X}}+\omega \cdot {\text{outcome}}_{\text{t}}^{\text{Y}}$$

$${\text{B}}_{\text{t}+1}^{\text{X}}=\left(1-\lambda \right)\cdot {\text{B}}_{\text{t}}^{\text{X}}+\tau \cdot \left(1-{\text{outcome}}_{\text{t}}^{\text{X}}\right)+\omega \cdot \left(1-{\text{outcome}}_{\text{t}}^{\text{Y}}\right)$$ (6)

For model fitting, we assume subjects are reporting the mean (μ) of this distribution (Eq 7).

$$\mu_{t}^{\text{X}}=\frac{{\text{A}}_{\text{t}}^{\text{X}}}{{\text{A}}_{\text{t}}^{\text{X}}+{\text{B}}_{\text{t}}^{\text{X}}}$$ (7)

And we derive a measure of uncertainty from the variance (σ^2^) of this distribution (Eq 8).

$$\sigma_{t}^{\text{2}\;\text{X}}=\frac{{\text{A}}_{\text{t}}^{\text{X}}\cdot {\text{B}}_{\text{t}}^{\text{X}}}{{\left({\text{A}}_{\text{t}}^{\text{X}}+{\text{B}}_{\text{t}}^{\text{X}}\right)}^{2}\cdot \left({\text{A}}_{\text{t}}^{\text{X}}+{\text{B}}_{\text{t}}^{\text{X}}+1\right)}$$ (8)

Finally, we tested an extension of the leaky beta model which features asymmetric updating, allowing alpha and beta (representing shock and no-shock outcomes respectively) to be updated at different rates by τ^+^ and τ^-^ respectively (Model 6, Eq 9).

$${\text{A}}_{\text{t}+1}^{\text{X}}=\left(1-\lambda \right)\cdot {\text{A}}_{\text{t}}^{\text{X}}+\tau^{+}\cdot {\text{outcome}}_{\text{t}}^{\text{X}}+\omega \cdot {\text{outcome}}_{\text{t}}^{\text{Y}}$$

$${\text{B}}_{\text{t}+1}^{\text{X}}=\left(1-\lambda \right)\cdot {\text{B}}_{\text{t}}^{\text{X}}+\tau^{-}\cdot \left(1-{\text{outcome}}_{\text{t}}^{\text{X}}\right)+\omega \cdot \left(1-{\text{outcome}}_{\text{t}}^{\text{Y}}\right)$$ (9)

All models were fit using an hierarchical Bayesian approach, assuming subject-level parameters are drawn from group-level distributions, with parameters estimated using Markov Chain Monte-Carlo sampling implemented in PyMC3 (https://docs.pymc.io/) with 2 chains of a 1000 sample initialisation followed by 3000 samples. Model comparison was performed using Watanabe-Akaike Information Criterion (WAIC) scores [56], which provides a goodness of fit measure for Bayesian models penalised according to the number of free parameters in the model.

As our aim was to examine visual attention during conventional forms of learning, we excluded any subjects who used a gambler’s fallacy-like heuristic strategy as opposed to a conventional learning strategy. We tested for this by first fitting both the standard Rescorla-Wagner model and the dual learning rate model, allowing the learning rate to vary between -1 and 1, and excluding subjects for whom any learning rate parameter was estimated as negative, indicating that probability estimates were reduced following a shock outcome and increased following a no-shock outcome.

### Eye tracking

Eye movements were recorded using an EyeLink 1000 eye tracker (SR Research) sampling at 1000hz. Participants were seated 73cm from the monitor and were not instructed to maintain fixation at any specific point to allow free viewing of the stimuli. Fixations were detected by the Eyelink system.

### Fixation analysis

All eye tracking data were analysed using Pyeparse (https://github.com/pyeparse/pyeparse). We focused our fixation analyses primarily on the pre-outcome phase of the trial, where subjects could see the stimuli on screen but had yet to receive any information about the outcomes on the current trial. This enabled us to investigate preferential preparatory attention prior to learning the outcome of the trial. Fixation bias was defined as the proportion of time spent fixating on one stimulus out of the total time spent fixating on both stimuli, providing an index of bias towards one stimulus over the other.

Value (represented by the subject’s shock probability estimates) and uncertainty (derived from the variance of our probabilistic computational model) were transformed into a bias index representing the difference between left and right stimuli (as seen on the screen at the pre-outcome phase), such that we predicted fixation bias from differences in relevant variables between stimuli. This allowed us to quantify the impact of learning-related influences on preferential visual attention. Relationships between behaviour and model-derived uncertainty were tested using beta regression with fixation duration predicted from value and uncertainty. As with the behavioural modelling, we used an hierarchical model, modelling fixation bias on a trial-by-trial basis and assuming subject-level parameters are drawn from a single group-level distribution. To aid convergence in this relatively complex multi-level model, regression coefficients were offset by an additional estimated parameter [57]. We excluded subjects from the analysis if they spent 80% of time fixating outside the stimuli during the pre-outcome phase. We also conducted a secondary analysis using the same procedure in the outcome phase of the trial, where subjects learn of the outcome for each stimulus. In all regression analyses, predictors were scaled to zero mean and unit variance to allow comparison between regression coefficients.

## Results

### Threat likelihood estimation is best described by a probabilistic model

Sixty-five subjects completed an aversive learning task where they reported the likelihood of receiving a shock from each of two stimuli displayed concurrently on screen, creating competition for attention. In each task block one of the stimuli had a stable shock probability and the other a variable shock probability, leading to a difference in uncertainty about shock probability between the two stimuli.

To quantify behaviour on the task, we fit a range of learning models to subjects’ ratings of upcoming shock probability. Two subjects were excluded for providing limited responses (i.e. exclusively using 0%, 50%, or 100%), and 12 were excluded for following a gambler’s fallacy-like heuristic as opposed to a learning strategy, choosing to decrease probability ratings after a shock (as determined by a negative estimated learning rate when fitting reinforcement learning models to the data).

**Fig 2:**
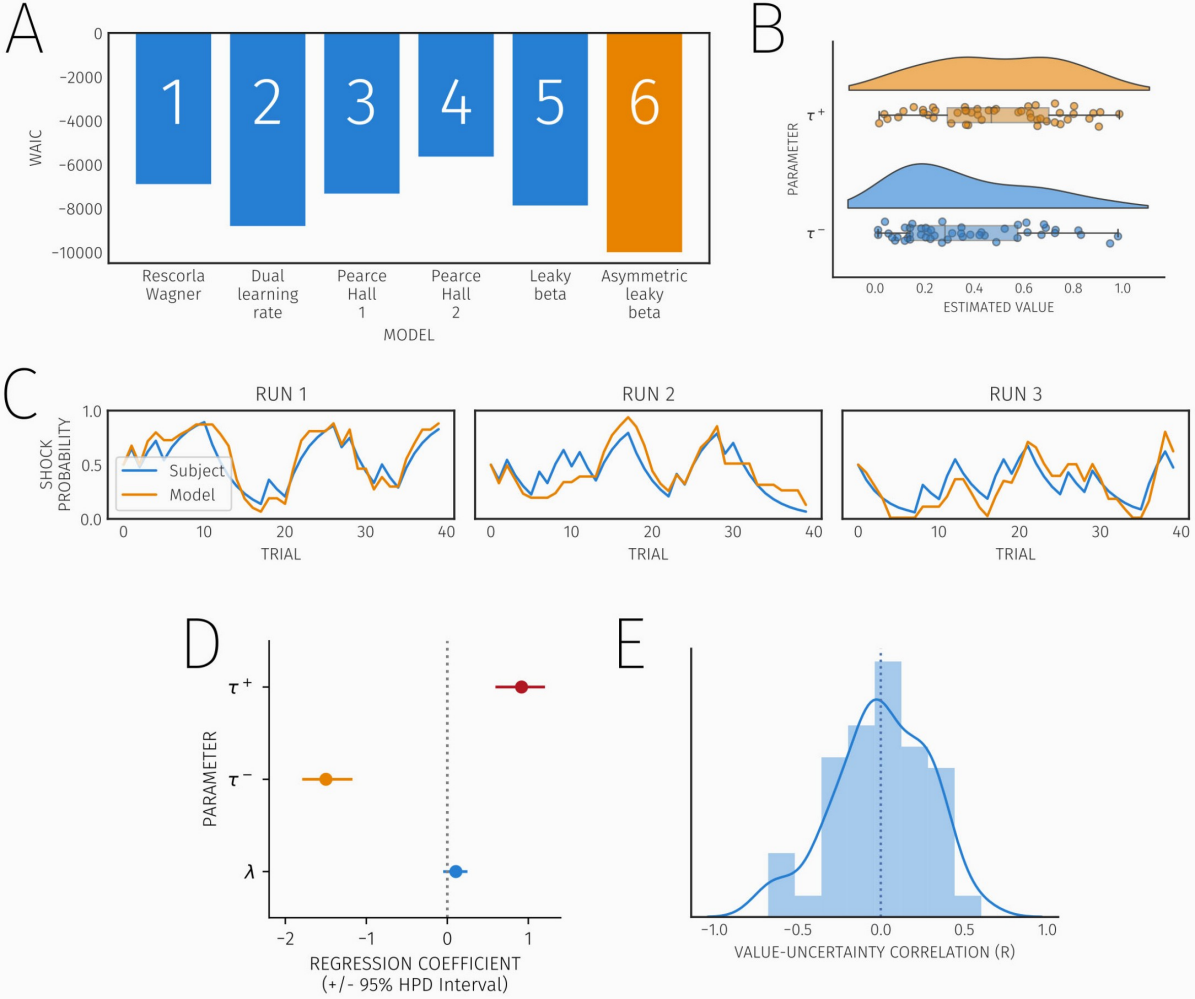
A) Results of model comparison (lower WAIC scores indicate better model fit) demonstrating model fit for the six evaluated models. The best fitting model (6) is shown in orange. B) Estimated values of τ^+^ and τ^-^, the parameters governing updates in response to shock and no-shock outcomes respectively, illustrating the bias in updating towards shock outcomes. C) True data and simulated shock probability estimate data from the asymmetric leaky beta model for an exemplar subject over three task blocks. This shows how well the model captures the pattern seen in the data. Blue lines represent the true data, while orange lines represent the simulated data from the model. D) Influence of model parameter values on mean shock probability estimates across subjects, red indicates the effect of τ^+^, the extent to which subjects update in response to shock outcomes, orange represents τ^-^, governing updates in response to no-shock outcomes, blue represents λ, the leak rate in the model. E) Histogram of subject-level correlations between value and variance, showing the dissociation between these two factors in the task. The dotted line represents the mean across subjects.

We tested six computational models of task behaviour including a family of reinforcement learning models and two heuristic Bayesian models that represented shock likelihood using beta distributions. According to the WAIC, a complexity-sensitive index of model fit, the best fitting model was an asymmetric leaky beta model (Model 6; Fig 2A), wherein subjects updated the parameters of a beta distribution differentially for shock and no-shock outcomes.

To further ensure that our model comparison was robust we evaluated model fit using cross validation, whereby each model was fit on three blocks and tested on the fourth block (using *R*^2^ as a measure of fit), across all four permutations of this split. The results of this analysis replicated the WAIC-based comparison (S1 Fig), providing convergent evidence that an asymmetric leaky beta model best explains our data, and suggesting that the WAIC provides a suitable approximation of cross-validated model fit.

Notably, subjects updated their estimates significantly more in response to shock (τ^+^) compared to no-shock outcomes (τ^-^) (*t* (47) = 7.09, *p* < 0.001), indicating a bias in learning (Fig 2B) such that subjects learned faster about negative (shock) compared to positive outcomes (a no shock outcome). To rule out a possibility that this bias was driven by shock probabilities being biased towards zero, we fit our model to blocks with the highest shock probability (with a combined mean probability of 50%, S2 Fig), yielding the same learning bias.

We also compared these models to variants where ω, representing the influence of the other stimulus (i.e. the stimulus shown on the other side of the screen), was fixed at zero, de facto removing it from the model. Model 6, which included a free ω parameter, remained the best fitting model while all models with this parameter fixed at zero performed worse than those with a free ω parameter (S1 Fig).

Across subjects the pattern seen in the estimated model parameters demonstrate an overall bias towards learning more from punishment compared to safety. To investigate whether individual differences in these parameter values were associated with individuals’ general shock expectancies, we used a Bayesian linear model to examine the contributions of three model parameters of interest (*τ*^*+*^, *τ*^*-*^, *λ*) to mean value estimates across trials (Fig 2D). This provides an approximate index of how threatening an individual perceives stimuli to be across the task. As expected, *τ*^*-*^ (the extent to which subjects update in response to no-shock outcomes) and *τ*^*+*^ (the extent to which subjects update in response to shock outcomes) had negative (mean regression coefficient = -1.49, 95% HPDI = -1.81, -1.17) and positive (mean regression coefficient = 0.91, 95% HPDI = 0.60, 1.23) effects on value estimates, respectively. In contrast *λ*, the decay rate, had no effect (mean regression coefficient = 0.10, 95% HPDI = -0.04, 0.26), suggesting that although decay contributes to learning it does not influence an individual’s tendency to under, or over-estimate threat likelihood. This supports the idea that individual differences in shock expectancy are explained by variation in two parameters of our learning model.

Our task design independently manipulated aversive value and uncertainty (represented as the variance of the beta distribution). To verify this manipulation was successful, we examined the correlation between value and uncertainty across trials for each subject (Fig 2E). The mean correlation across subjects was -0.001 (SD = 0.28), with a one sample t-test confirming these correlations did not differ significantly from zero (*t* (48) = 0.028, *p* = .98), consistent with the independence of these quantities.

### Visual selective attention is guided by value but not uncertainty

To examine the dependence of visual attention on learning-related variables, we used trial-wise Bayesian beta regression to predict a bias in fixation duration for the pre-outcome phase of the trial (defined as the proportion of all stimulus-directed fixation time spent fixating on one stimulus) as a function of difference in value (represented by subjective shock probability estimates) and model-derived uncertainty between the two stimuli. This time period was chosen to allow examination of preparatory attention when an outcome is expected, prior to actual outcome receipt. We used an hierarchical approach that estimates effects on a trial-wise basis within subject, assuming the parameters governing these effects are drawn from common group-level distributions. We excluded two subjects at this stage as they spent >80% of fixation time outside the stimuli of interest.

**Fig 3:**
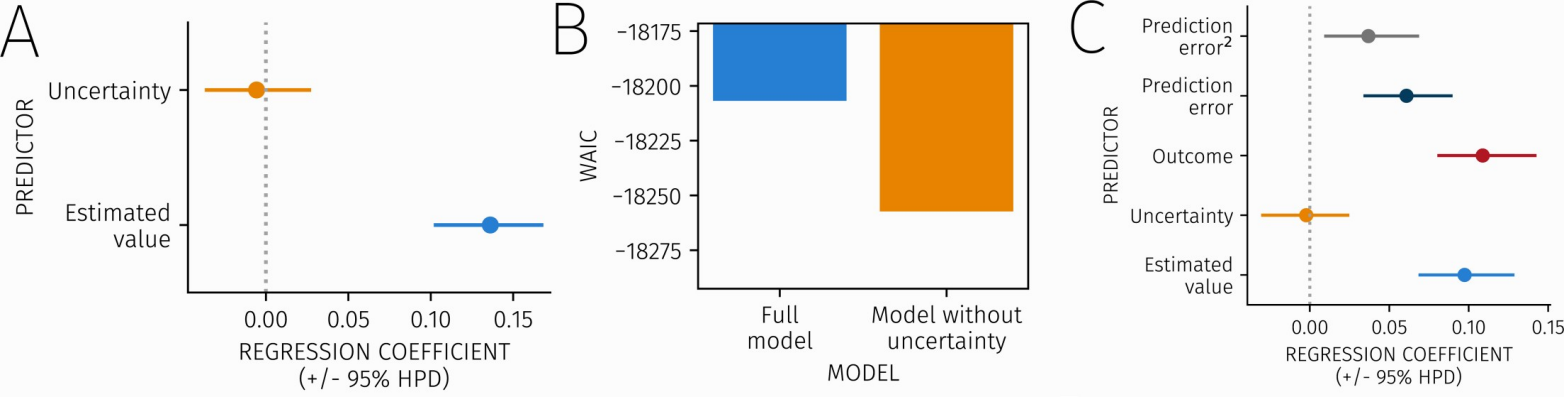
A) Parameter estimates from the beta regression model predicting fixation bias from value and uncertainty. Orange represents the effect of uncertainty, while blue represents the effect of value on fixation bias. B) Results of model comparison, comparing the full beta regression model including an effect of a shock to a simpler model without an effect of uncertainty. The winning model is shown in orange. C) Parameter estimates from a model predicting fixation bias during the outcome phase (when subjects are shown the outcome for each stimulus) from value, uncertainty, outcome (shock or no shock), prediction error, and squared prediction error.

Estimates from the beta regression model (shown in Fig 3A and Table 1) indicate that value had a positive effect on fixation duration, with 100% of the posterior density for the regression coefficient governing the influence of value above zero. In contrast, the *β* parameter for uncertainty was near zero, with low uncertainty around this estimate. This suggests that aversive value, but not uncertainty, influenced visual selective attention.

**Table 1:** Parameter estimates for regression models predicting fixation bias in the pre-outcome and outcome phases of the trial. Mean *β* represents the mean estimated *β* value of the predictor in the regression model, while the 2.5% and 97.5% HPDI values represent the upper and lower interval on the 95% highest posterior density of the posterior distribution over the estimated parameter values.

Trial phase	Predictor	Regression coefficient	2.5% HPDI	97.5% HPDI
Pre-outcome	Value	0.13	0.11	0.17
	Uncertainty	-0.004	-0.04	0.03
Outcome	Value	0.10	0.07	0.13
	Uncertainty	-0.004	-0.03	0.03
	Outcome	0.10	0.07	0.13
	Prediction error	0.07	0.04	0.10
	Prediction error^2^	0.03	0.002	0.06

To probe further a surprising null effect for uncertainty, we formally compared WAIC scores between our original beta regression model and a simpler model that excluded an effect of uncertainty (Fig 3B). Accounting for model complexity, a model without an uncertainty term provided the best fit to the fixation bias data, providing additional evidence against an effect of uncertainty on fixation.

We explored next the relationship between value and uncertainty and two other task variables, namely the duration of first fixation bias (defined as the difference between first fixation duration for the two stimuli) and the location of the first fixation during the pre-outcome phase. Bayesian regression analyses found no effect of value or uncertainty on either of these task metrics, with all 95% HPD intervals including zero. Replicating this analysis using model-derived value estimates produced the same pattern of results (S4 Fig). Finally, to provide a less model-based approximation of this analysis, we repeated our original analysis but replaced model-derived uncertainty with the magnitude of the prediction error on the previous trial. Although prediction error had a greater effect on fixation than model-derived uncertainty, as with the model-based analysis the 95% HPD interval for the prediction error effect crossed zero (S4 Fig).

Finally, we asked whether learning influenced attention during the outcome phase, when subjects learn about the trial outcome for each stimulus (Fig 3C and Table 1). As in the pre-outcome phase, we observed effects of value but again found no effect of pre-outcome uncertainty. In fact, we found effects in response to three variables, outcome (shock or no shock), signed prediction error (the difference between observed outcome and predicted shock probability), and squared prediction error (representing an unsigned prediction error), all of which showed positive effects on fixation duration.

### Aversive value estimates are influenced by visual attention

Having established that aversive learning impacts attention we asked next whether this relationship is bidirectional, such that learning itself is impacted by visual attention. First, using trial-wise hierarchical regression, we examined how attention at the outcome phase of the previous trial influenced probability estimates on the current trial. Here, the target of our regression was estimation error (the difference between reported and true shock probability), representing how much subjects’ estimates differed from the true shock probabilities. As regressors we used true shock probability for both stimuli, the outcome of the trial, and the proportion of time spent fixating the stimulus on the previous trial. This allowed us to measure effects of visual attention on probability estimation over and above effects of outcome and stimulus value itself. This analysis revealed negative effects of the true probability of both the currently estimated stimulus (mean regression coefficient = -0.18, 95% HPDI = -0.18, -0.18) and the alternative stimulus (mean regression coefficient = -0.02, 95% HPDI = -0.03, -0.02), such that shock probability was overestimated when true shock probability was low, and underestimated when it was high (S5 Fig). Importantly, there was also a small but consistent positive effect of fixation duration such that subjects overestimated shock likelihood when they attended stimuli for a longer duration on the previous trial (mean regression coefficient = 0.017, 95% HPDI = 0.013, 0.021).

An influence of fixation on probability estimates could arise out of multiple mechanisms. One possibility is that attending to a stimulus increases the degree to which its value is updated. This would exaggerate an existing tendency to update estimates faster following punishment, resulting in a greater overestimation of shock probability. Alternatively, this type of influence could take the form of a general bias, whereby stimuli that are attended gain additional value. To provide a more precise formalisation of how attention influences the learning process we fitted two additional variants of our winning behavioural model. The first of these (model 6A; Eq 10) biased the rate of updating for each stimulus based on the proportion of stimulus fixation time during the outcome phase of the previous trial, thereby biasing the update process itself. The degree of weighting (π) is itself modulated by an additional free parameter *γ*. In addition, we allowed the influence of the other stimulus to be weighted by the proportion of time spent looking at that stimulus. Given the small magnitude of the other stimulus’s influence, we chose to let its effect be fully weighted by fixation rather than having the degree of fixation influence modulated by an additional parameter, as additional modulating parameters here would be challenging to estimate accurately.

$$\pi_{\text{t}}^{\text{X}}={\;\text{fixation}}_{\text{t}}^{\text{X}}\cdot \gamma +\left(1-\gamma \right)$$

$${\text{A}}_{\text{t}+1}^{\text{X}}=\left(1-\lambda \right)\cdot {\text{A}}_{\text{t}}^{\text{X}}+\tau^{+}\cdot \pi_{\text{t}}^{\text{X}}\cdot {\text{outcome}}_{\text{t}}^{\text{X}}+\omega \cdot {\text{fixation}}_{\text{t}}^{\text{Y}}\cdot {\text{outcome}}_{\text{t}}^{\text{Y}}$$

$${\text{B}}_{\text{t}+1}^{X}=\left(1-\lambda \right)\cdot {\text{B}}_{\text{t}}^{\text{X}}+\tau^{-}\cdot \pi_{\text{t}}^{\text{X}}\cdot \left(1-{\text{outcome}}_{\text{t}}^{\text{X}}\right)+\omega \cdot {\text{fixation}}_{\text{t}}^{\text{Y}}\cdot \left(1-{\text{outcome}}_{\text{t}}^{\text{Y}}\right)$$ (10)

Here the proportion of time spent fixating on stimulus X or Y is represented by fixation^X^ or fixation^Y^ respectively. For comparison, we took a second model variant (model 6B; Eq 11) which added value to each stimulus dependent on how much it was fixated, increasing the alpha parameter by an amount equal to the fixation proportion for the stimulus, weighted by an additional free parameter *θ*. Rather than modulating update rates, this model biases value estimates such that attended stimuli have a higher value in a manner similar to models that incorporate choice perseveration through an increase in the value of chosen options [58].

$${\text{A}}_{\text{t}+1}^{\text{X}}=\left(1-\lambda \right)\cdot A_{\text{t}}^{\text{X}}+\tau^{+}\cdot {\text{outcome}}_{\text{t}}^{\text{X}}+\omega \cdot {\text{fixation}}_{\text{t}}^{\text{Y}}\cdot {\text{outcome}}_{\text{t}}^{\text{Y}}+\theta \cdot {\text{fixation}}_{\text{t}}^{\text{X}}$$

$${\text{B}}_{\text{t}+1}^{\text{X}}=\left(1-\lambda \right)\cdot {\text{B}}_{\text{t}}^{\text{X}}+\tau^{-}\cdot \left(1-{\text{outcome}}_{\text{t}}^{\text{X}}\right)+\omega \cdot {\text{fixation}}_{\text{t}}^{\text{Y}}\cdot \left(1-{\text{outcome}}_{\text{t}}^{\text{Y}}\right)$$ (11)

**Fig 4:**
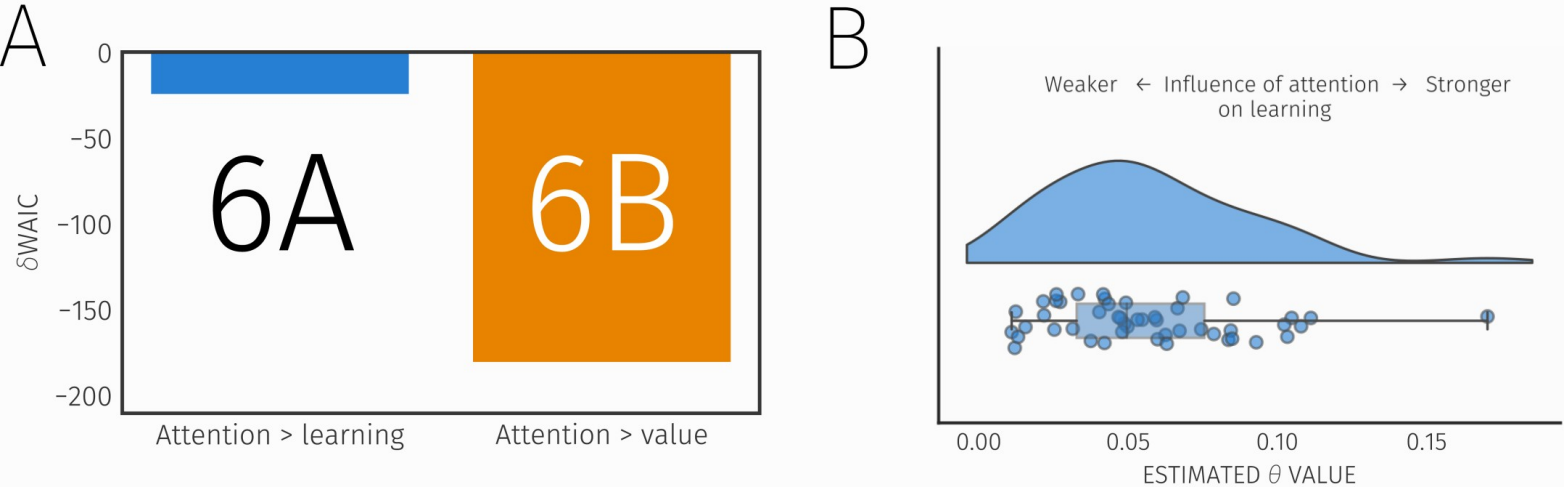
A) Results of model comparison, showing WAIC scores (relative to the original best fitting model) for two alternative models that incorporated effects of attention. The best fitting model (Model 6B), incorporating an effect of attention on learning through biasing value, is shown in orange, along with the original best fitting model (Model 6) and an alternative model (Model 6A) where attention modulated updates, B) Distribution of θ values (reflecting the influence of attention on learning) across subjects.

These two models allowed us to determine whether longer fixations increased probability estimates by modulating updates, or by simply biasing positively the value of a stimulus. Model comparison demonstrated Model 6B, a model that allowed attention to bias value estimates, provided the best fit to the data (Fig 4A). This supports an idea that visual attention guides learning by biasing value estimates upwards. Examining the estimated values of the free parameter θ, which governs the influence of attention on learning, showed all subjects had non-zero estimates for this parameter (Fig 4B).

## Discussion

By manipulating both aversive value and uncertainty, we show that selective visual attention is guided by subjective estimates of value, but not uncertainty, during aversive prediction and learning. Moreover, this relationship between attention and learning is bidirectional, such that attention also guides learning. To our knowledge, this is the first study that has examined an influence of subjective value and uncertainty on visual attention in the context of aversive learning. The findings highlight a complex interplay between perceptual attention and aversive learning.

Our results offer some support for theoretical accounts of the behavioural role of attention in competitive associative learning. The Mackintosh and Pearce-Hall models [3,4] emphasise a correlation with reinforcement (often in the form of expected value) and uncertainty respectively in the allocation of attention, albeit with respect to associability (rather than visual selective attention), in contexts requiring learning and prediction for competing stimuli. Previous empirical work on competitive perceptual associative learning provide support for both models [16,25,39]. Our results support predictions of the former model, in particular, but in a subtly different context where subjects make predictions from, and learn about, multiple stimuli concurrently. This finding is of considerable interest as uncertainty is often invoked as providing a normative explanation for attentional allocation during learning [59,60]. Our results suggest that in aversive environments when faced with the task of learning about multiple stimuli concurrently, human subjects may not always adapt their attention based on an ongoing estimate of uncertainty.

A potential explanation for the observed effect of value evokes the notion of Pavlovian biases, which engender automatic orienting towards stimuli with high aversive value, a so-called “risk assessment” behaviour that is well documented in rodents and humans when faced with threat [32–35]. Conversely, aversive stimuli often prompt avoidance responses that are difficult to overcome [30,45]. The former appears to dominate in that, although our task did not allow subjects to actually avoid shock, it is possible that attention is automatically and involuntarily allocated to targets with high aversive value so as to facilitate avoidance. However, this is a speculative interpretation that requires validation in an experiment designed to specifically test non-goal directed orienting of attention. Although we observed no effect of ongoing uncertainty estimates, in the phase of the experiment where subjects learned about the outcome of a trial, we observed effects of both signed and unsigned prediction errors, replicating previous work in non-aversive tasks [27]. This suggests that attention at the point of learning is influenced by surprise, itself an indication of future uncertainty. This points towards a complex relationship between momentary markers of uncertainty such as prediction errors, ongoing estimation uncertainty, and attention, whereby attention is influenced by momentary indications of future uncertainty but not by the general current level of uncertainty.

Our second key result is a bidirectional relationship between learning and attention, whereby stimuli that are the focus of visual attention during learning are given heightened value estimates, being perceived as more threatening. This implies visual attention is not simply a by-product of the learning process but actively contributes to learning about aversive stimuli. This finding builds on a literature demonstrating that visual attention biases value-based decisions [6,7]. In addition, it echoes a recent study of reward-guided learning and decision making [28], showing that stimuli that were attended during decision making were learned about faster than unattended stimuli. We show, for the first time to the best of our knowledge, that attention influences value estimates during aversive learning, suggesting that value estimation in both reward and punishment domains is subject to influences from visual attention. However, an important observation in our study, and one which contrasts with this previous work, is that this effect does not occur through modulation of updates but instead biases value estimates upwards. This result is reminiscent of effects predicted by attentional drift diffusion models [6,7,17], suggesting that enhanced attention leads to inflated value estimates.

Behaviour in our task was described best by a probabilistic model, as opposed to reinforcement learning models. This is consistent with subjects maintaining approximate probability distributions over aversive events, and adapting their learning accordingly. For example, in our model, updates have a relatively diminished effect on overall probability estimates as evidence is accumulated, and variance of the distribution reduces. As expected, individual differences in update parameters in this model were associated with mean shock probability ratings, allowing us to explain individual variability in shock expectancy through differences in learning processes. We note others have shown that probabilistic models can provide a superior fit to data than reinforcement learning models in aversive [49,55] and reward based learning tasks [54]. Although neuroimaging evidence suggests that the brain maintains mean and variance estimates [61–64], providing a neural implementation for such a probabilistic model, we make no claims here regarding implementation. Instead, our main aim was to use the model to examine a link between uncertainty estimation and attention.

Our findings have implications for understanding cognitive biases in pathological anxiety. A large literature suggests that individuals with anxiety disorders, as well as individuals high in trait anxiety, express an attentional bias towards threatening stimuli, such as faces displaying negatively-valenced emotions [40,41]. There is also evidence suggesting this bias plays a causal role in symptom development [42]. However, the origin of this attentional bias has thus far remained unclear. Our results suggest that threat-related attentional biases might be a downstream effect of dysfunctional learning leading to exaggerated value estimates (i.e. a subjective overestimation of threat probability). Additionally, our observation of an effect of attention on learning suggests a possible self-reinforcing cycle, whereby inappropriate learning about the environment leads to biased attention and, in turn, inaccurate value estimates.

Our task addresses a different question to that of many prior studies of selective attention during learning. These studies typically focus on the function of selective attention in assigning a single outcome to multiple competing stimuli (24,25,27,39). Here, by contrast, multiple stimuli are learned about concurrently, allowing us to address the question of how limited visual attentional resources are guided by learned value, and vice versa. We expected that attention and learning would both be affected by this concurrency, with both optimising and heuristic influences. From an optimising perspective, in the face of limited time and processing resources, attention and learning should focus on the stimuli that are most important to learn about (i.e. those where maintaining an accurate value estimate has the greatest effect on the loss function). Even though the experiment was not tachistoscopic, subjects had only limited time for estimation and updating. In terms of heuristics, it has been demonstrated that human subjects show perceptual attentional biases towards threat-related stimuli, even during tasks where there is no need for prioritised attention (40). Consequently, in our task we expected similar biases would arise as a result of stimuli acquiring aversive value. Of course, factors that are sub-optimal in this task may be optimal in other tasks.

One limitation of our study is our inability to examine choice behaviour, linked previously to attentional processes in the reward domain [28,65]. We chose a design requiring subjects estimate shock probabilities for two stimuli concurrently based on two considerations. Firstly, continuous probability estimates (as opposed to binary choices) provide arguably richer data, producing better model fits and more accurate model-derived estimates of uncertainty. Secondly, the design limits avoidance-related problems. For instance, if subjects choose to avoid one stimulus this would subsequently be unattended simply because it is irrelevant for the current trial. However, the probability estimation period of this task is not well suited to attention-related analyses, as attention is largely related to motor actions required to provide probability estimates.

With regard to attention, one factor left uncontrolled for that might influence our results is the choice of stimuli: circles and squares were used as signals of safety and shocks respectively across all subjects. While we consider it unlikely that there was differential attention towards either of these non-salient stimuli we cannot exclude potential preferential attention to either of these shapes. A further limitation of our design is that it does not allow us to cleanly disentangle the roles of covert and overt attention. It is possible that overt attention may be involved in ongoing value updating processes after the initial outcome presentation. Additionally, it is possible that any influence of estimation uncertainty may only become apparent in contexts where there is a greater need to allocate attention preferentially, such as in situations involving perceptual uncertainty.

In summary, we demonstrate a bidirectional relationship between learning and attention within aversive environments, where learning guides attention and in turn attention guides learning. The findings have implications for understanding how aversive value is learned and hints at an important role for learning in the development of pathological threat-related attentional biases in clinical anxiety.

## Supporting information

S1 Fig Results of model comparison using cross-validation. Models were fit on three out of four blocks of the task and the resulting parameter values were used to simulate data for the fourth block. The fit of this simulated data to the true behaviour on the left-out block was quantified using *R*^2^ providing an index of how well our model captured behaviour that was not used for fitting. This was repeated for every combination of blocks, resulting in four folds of cross-validation, and the average across all folds is reported here. This demonstrates that the asymmetric leaky beta model (model 6) fits best according to both the WAIC and cross-validation metrics and provides evidence that the WAIC provides an accurate approximation of cross-validated model fit.
(EPS)

S2 Fig Values for parameters governing updates in response to shock (τ^+^) and no shock (τ^-^) outcomes for only the 45% and 55% shock probability blocks, demonstrating that a bias in learning is still present even in blocks where the overall shock probability is not biased towards zero. (EPS)

S3 Fig A) Model comparison demonstrating fit of models including a free ω term (governing the amount to which the shock probability is updated for stimulus X when stimulus Y is shocked) relative to models where ω is fixed at zero (i.e. no influence of the other stimulus). Models including this stimulus interference perform better according to the WAIC scores, suggesting that there is a degree of interference between stimuli, and the best performing model (the asymmetric leaky beta model, model 6) with a free ω parameter is the best fitting model overall. B) Behavioural evidence for stimulus interference, represented as the difference between updates for stimulus X when Y was shocked and updates for stimulus X when Y was not shocked. There is a large degree of inter-subject variability in this interference effect, with some subjects showing a positive effect and others showing a negative effect. Permutation testing on the absolute value of this effect (i.e. looking at whether there was any interference effect regardless of its direction), randomly shuffling X and Y stimulus labels, showed that the level of interference was significantly greater than would be expected under the null hypothesis (p = .01). C) Relationship between the level of behavioural interference (as shown in panel B) and the estimated value of ω in the winning model, showing that subjects with greater interference effects at a behavioural have higher estimated interference parameter values (R = .63, p < .001).
(EPS)

S4 Fig A) Split-half reliability for regression coefficients in the model predicting fixation from value and uncertainty. The values of the regression coefficients are near-identical in both halves of the experiment, suggesting that these are robust and reliable effects. B) Results of a model replacing reported value with model-derived value. The result of value is similar to that from the original model, although marginally weaker (regression coefficient = .134 in the original model vs .118 when using model-derived value), as would be expected when using a less direct measure. C) A similar model replacing model-derived uncertainty with absolute prediction error magnitude on the previous trial, as a model-free approximation of uncertainty. This demonstrates a weak effect of prediction error magnitude (regression coefficient = 0.03), although the 95% HPD intervals do include zero (-0.0003–0.062).
(EPS)

S5 Fig Mis-estimation of shock probabilities in stable and volatile blocks, showing that subjects tend to overestimate low probabilities and underestimate high shock probabilities, an effect that is driven by the volatile blocks. Individual lines represent individual subjects, while bold lines represent the group mean.
(EPS)

S6 FigModel fit for models incorporating continuous and binary weighting of fixation relative to the original model without an effect of fixation, demonstrating that the model incorporating a continuous effect of attention on value provides the best fit.The continuous models are described in the main manuscript. Updates for the binary version of model 5A proceed as follows:
$$\pi^{\text{X}}=\begin{array}{cc} 1 & {\text{if}\;\text{fixation}}^{\text{X}}>{\text{fixation}}^{\text{Y}}\\ 1-\left({\text{fixation}}^{\text{X}}-{\text{fixation}}^{Y}\right)\cdot \gamma & {\text{if}\;\text{fixation}}^{\text{X}}<{\text{fixation}}^{\text{Y}} \end{array}$$
$$A_{\text{t}+1}^{\text{X}}=\left(1-\lambda \right)\cdot A_{\text{t}}^{\text{X}}+\tau^{+}\cdot \pi^{\text{X}}\cdot {\text{outcome}}^{\text{X}}+\omega \cdot {\text{fixation}}^{\text{Y}}\cdot {\text{outcome}}^{\text{Y}}$$
$B_{\text{t}+1}^{\text{X}}=\left(1-\lambda \right)\cdot B_{\text{t}}^{\text{X}}+\tau^{-}\cdot \pi^{\text{X}}\cdot \left(1-{\text{outcome}}^{\text{X}}\right)+\omega \cdot {\text{fixation}}^{\text{Y}}\cdot \left(1-{\text{outcome}}^{\text{Y}}\right)$While model the binary variant of model 5B updates as follows:
$$\alpha_{\text{t}+1}^{\text{A}}=\left(1-\lambda \right)\cdot \alpha_{\text{t}}^{\text{A}}+\tau^{+}\cdot {\text{outcome}}^{\text{A}}+\omega \cdot {\text{outcome}}^{\text{B}}+\text{max}\left(0,{\text{fixation}}^{\text{A}}-{\text{fixation}}^{\text{B}}\right)\cdot \theta$$
$\beta_{\text{t}+1}^{\text{A}}=\left(1-\lambda \right)\cdot \beta_{\text{t}}^{\text{A}}+\tau^{-}\cdot \left(1-{\text{outcome}}^{\text{A}}\right)+\omega \cdot \left(1-{\text{outcome}}^{\text{B}}\right)$(EPS)

S7 Fig Results of analysis predicting anxiety scores from model parameters and learning-related variables. We investigated relationships between state and trait anxiety, measured using the State-Trait Inventory for Cognitive and Somatic Anxiety, and learning-related variables (model parameters, mean model-derived uncertainty across trials, and mean reported shock probability) using Bayesian linear regression implemented in Bambi (https://github.com/bambinos/bambi). Although we titrated shock intensity to ensure similar subjective shock unpleasantness across subjects, we also included reported shock unpleasantness (rated post-experiment) as a covariate to control for any potential confounding effects of residual differential sensitivity to the shocks that remained after this procedure. All questionnaire measures were completed immediately after completing the experiment. Each point in the plot represents the beta value of the predictor in a GLM predicting either trait (blue) or state (orange) anxiety, and the error bars represent the 95% highest posterior density intervals. Due to an error in recording of questionnaire data, two subjects were excluded from this analysis. None of our explanatory variables were significant predictors of trait anxiety. However, we observed an unexpected positive relationship between the parameter governing the rate of updating in response to no-shock outcomes (τ^-^) and state anxiety (mean *β* = 0.67, 95% HPDI = 0.10, 1.18), wherein more anxious individuals showed enhanced learning about safety. There were no associations between other model parameters and state or trait anxiety, while we observed trend-level effects of anxiety on value and uncertainty estimates, with more anxious individuals having lower value and uncertainty. However, the highest posterior density intervals for these effects included zero, indicating that we cannot be confident that these effects are meaningful. Thus, we observe an unexpected relationship between our model parameter governing updates in response to no-shock outcomes and state anxiety, whereby more anxious individuals learned faster from the absence of shock than those with low anxiety. Additionally, there was a trend towards more anxious individuals underestimating shock probability, possibly resulting from their tendency to learn faster about safety. This observation runs counter to existing work. Given the likely small effect size for any relationship between non-clinical trait anxiety and task-related parameters, as well as known issues in accurately estimating such effects in small samples, it is possible this finding is simply a mis-estimation of a true association with a small effect size in a different direction. Further work involving larger samples, potentially availing of online mass data collection, or clinical samples will be required to investigate more deeply the precise relationships between aversive learning and anxiety.
(EPS)

## References

[1] Broadbent DE. Perception and communication. Elmsford, NY, US: Pergamon Press; 1958 10.1037/10037-000

[2] Treisman AM. Strategies and models of selective attention. Psychological Review. 1969;76: 282–299. 10.1037/h0027242 4893203

[3] Pearce JM, Hall G. A model for Pavlovian learning: Variations in the effectiveness of conditioned but not of unconditioned stimuli. Psychological Review. 1980;87: 532–552. 10.1037/0033-295X.87.6.532 7443916

[4] Mackintosh NJ. A theory of attention: Variations in the associability of stimuli with reinforcement. Psychological Review. 1975;82: 276–298.

[5] Rogers RD, Andrews TC, Grasby PM, Brooks DJ, Robbins TW. Contrasting Cortical and Subcortical Activations Produced by Attentional-Set Shifting and Reversal Learning in Humans. Journal of Cognitive Neuroscience. 2000;12: 142–162. 10.1162/089892900561931 10769312

[6] Krajbich I, Rangel A. Multialternative drift-diffusion model predicts the relationship between visual fixations and choice in value-based decisions. PNAS. 2011;108: 13852–13857. 10.1073/pnas.1101328108 21808009 PMC3158210

[7] Krajbich I, Armel C, Rangel A. Visual fixations and the computation and comparison of value in simple choice. Nat Neurosci. 2010;13: 1292–1298. 10.1038/nn.2635 20835253

[8] Dayan P, Zemel RS. Statistical models and sensory attention. 1999 Ninth International Conference on Artificial Neural Networks ICANN 99 (Conf Publ No 470). 1999. pp. 1017–1022 vol.2. 10.1049/cp:19991246

[9] Dayan P, Kakade S, Montague PR. Learning and selective attention. Nat Neurosci. 2000;3: 1218–1223. 10.1038/81504 11127841

[10] Tsotsos JK. Analyzing vision at the complexity level. Behavioral and Brain Sciences. 1990;13: 423–445. 10.1017/S0140525X00079577

[11] Zhaoping L. Understanding Vision: Theory, Models, and Data. Oxford, United Kingdom; New York, NY, United States of America: Oxford University Press; 2014.

[12] Sutton RS. Gain adaptation beats least squares. Proceedings of the 7th Yale workshop on adaptive and learning systems. 1992.

[13] Anderson BA. The attention habit: how reward learning shapes attentional selection. Ann NY Acad Sci. 2016;1369: 24–39. 10.1111/nyas.12957 26595376

[14] Anderson BA, Laurent PA, Yantis S. Value-driven attentional capture. PNAS. 2011;108: 10367–10371. 10.1073/pnas.1104047108 21646524 PMC3121816

[15] Chelazzi L, Eštočinová J, Calletti R, Gerfo EL, Sani I, Libera CD, et al Altering Spatial Priority Maps via Reward-Based Learning. J Neurosci. 2014;34: 8594–8604. 10.1523/JNEUROSCI.0277-14.2014 24948813 PMC6608215

[16] Pelley MEL, Pearson D, Porter A, Yee H, Luque D. Oculomotor capture is influenced by expected reward value but (maybe) not predictiveness. The Quarterly Journal of Experimental Psychology. 2017;0: 1–46. 10.1080/17470218.2017.1313874 28375688

[17] Krajbich I, Lu D, Camerer C, Rangel A. The Attentional Drift-Diffusion Model Extends to Simple Purchasing Decisions. Front Psychol. 2012;3 10.3389/fpsyg.2012.00193 22707945 PMC3374478

[18] Roesch MR, Esber GR, Li J, Daw ND, Schoenbaum G. Surprise! Neural correlates of Pearce–Hall and Rescorla–Wagner coexist within the brain. European Journal of Neuroscience. 2012;35: 1190–1200. 10.1111/j.1460-9568.2011.07986.x 22487047 PMC3325511

[19] Hall G, Pearce JM. Latent inhibition of a CS during CS-US pairings. J Exp Psychol Anim Behav Process. 1979;5: 31–42. 528877

[20] Baxter MG, Holland PC, Gallagher M. Disruption of Decrements in Conditioned Stimulus Processing by Selective Removal of Hippocampal Cholinergic Input. J Neurosci. 1997;17: 5230–5236. 10.1523/JNEUROSCI.17-13-05230.1997 9185560 PMC6573295

[21] Behrens TEJ, Woolrich MW, Walton ME, Rushworth MFS. Learning the value of information in an uncertain world. Nat Neurosci. 2007;10: 1214–1221. 10.1038/nn1954 17676057

[22] Boll S, Gamer M, Gluth S, Finsterbusch J, Büchel C. Separate amygdala subregions signal surprise and predictiveness during associative fear learning in humans. European Journal of Neuroscience. 2012;37: 758–767. 10.1111/ejn.12094 23278978

[23] Li SSY, McNally GP. The conditions that promote fear learning: Prediction error and Pavlovian fear conditioning. Neurobiology of Learning and Memory. 2014;108: 14–21. 10.1016/j.nlm.2013.05.002 23684989

[24] Le Pelley ME, Beesley T, Griffiths O. Overt attention and predictiveness in human contingency learning. Journal of Experimental Psychology: Animal Behavior Processes. 2011;37: 220–229. 10.1037/a0021384 21319915

[25] Beesley T, Nguyen KP, Pearson D, Pelley MEL. Uncertainty and predictiveness determine attention to cues during human associative learning. The Quarterly Journal of Experimental Psychology. 2015;68: 2175–2199. 10.1080/17470218.2015.1009919 25832459

[26] Pelley MEL, Haselgrove M, Esber GR. Modeling attention in associative learning: Two processes or one? Learn Behav. 2012;40: 292–304. 10.3758/s13420-012-0084-4 22927002

[27] Wills AJ, Lavric A, Croft GS, Hodgson TL. Predictive Learning, Prediction Errors, and Attention: Evidence from Event-related Potentials and Eye Tracking. Journal of Cognitive Neuroscience. 2007;19: 843–854. 10.1162/jocn.2007.19.5.843 17488208

[28] Leong YC, Radulescu A, Daniel R, DeWoskin V, Niv Y. Dynamic Interaction between Reinforcement Learning and Attention in Multidimensional Environments. Neuron. 2017;93: 451–463. 10.1016/j.neuron.2016.12.040 28103483 PMC5287409

[29] Armel KC, Beaumel A, Rangel A. Biasing simple choices by manipulating relative visual attention. Judgment and Decision Making. 2008;3: 396–403.

[30] Guitart-Masip M, Huys QJM, Fuentemilla L, Dayan P, Duzel E, Dolan RJ. Go and no-go learning in reward and punishment: Interactions between affect and effect. NeuroImage. 2012;62: 154–166. 10.1016/j.neuroimage.2012.04.024 22548809 PMC3387384

[31] Guitart-Masip M, Fuentemilla L, Bach DR, Huys QJM, Dayan P, Dolan RJ, et al Action Dominates Valence in Anticipatory Representations in the Human Striatum and Dopaminergic Midbrain. J Neurosci. 2011;31: 7867–7875. 10.1523/JNEUROSCI.6376-10.2011 21613500 PMC3109549

[32] Blanchard DC. Stimulus, environmental, and pharmacological control of defensive behaviors Learning, motivation, and cognition: The functional behaviorism of Robert C Bolles. Washington, DC, US: American Psychological Association; 1997 pp. 283–303. 10.1037/10223-014

[33] Takahashi LK, Nakashima BR, Hong H, Watanabe K. The smell of danger: A behavioral and neural analysis of predator odor-induced fear. Neuroscience & Biobehavioral Reviews. 2005;29: 1157–1167. 10.1016/j.neubiorev.2005.04.008 16095694

[34] Van der Poel AM. A note on “stretched attention,” a behavioural element indicative of an approach-avoidance conflict in rats. Animal Behaviour. 1979;27: 446–450. 10.1016/0003-3472(79)90181-7

[35] Perkins AM, Ettinger U, Davis R, Foster R, Williams SCR, Corr PJ. Effects of Lorazepam and Citalopram on Human Defensive Reactions: Ethopharmacological Differentiation of Fear and Anxiety. J Neurosci. 2009;29: 12617–12624. 10.1523/JNEUROSCI.2696-09.2009 19812336 PMC6665108

[36] Austin AJ, Duka T. Mechanisms of attention for appetitive and aversive outcomes in Pavlovian conditioning. Behavioural Brain Research. 2010;213: 19–26. 10.1016/j.bbr.2010.04.019 20412818

[37] Van Damme S, Crombez G, Hermans D, Koster EHW, Eccleston C. The role of extinction and reinstatement in attentional bias to threat: A conditioning approach. Behaviour Research and Therapy. 2006;44: 1555–1563. 10.1016/j.brat.2005.11.008 16375852

[38] Schmidt LJ, Belopolsky AV, Theeuwes J. Potential threat attracts attention and interferes with voluntary saccades. Emotion. 2015;15: 329–338. 10.1037/emo0000041 25527964

[39] Hogarth L, Dickinson A, Austin A, Brown C, Duka T. Attention and expectation in human predictive learning: The role of uncertainty. The Quarterly Journal of Experimental Psychology. 2008;61: 1658–1668. 10.1080/17470210701643439 18942033

[40] Armstrong T, Olatunji BO. Eye tracking of attention in the affective disorders: A meta-analytic review and synthesis. Clinical Psychology Review. 2012;32: 704–723. 10.1016/j.cpr.2012.09.004 23059623 PMC3556338

[41] Bar-Haim Y, Lamy D, Pergamin L, Bakermans-Kranenburg MI, van IJzendoorn MH, yair1@post.tau.ac.il Threat-Related Attentional Bias in Anxious and Nonanxious Individuals: A Meta-Analytic Study. Psychological Bulletin. 2007;133: 1–24. 10.1037/0033-2909.133.1.1 17201568

[42] MacLeod C, Rutherford E, Campbell L, Ebsworthy G, Holker L. Selective attention and emotional vulnerability: Assessing the causal basis of their association through the experimental manipulation of attentional bias. Journal of Abnormal Psychology. 2002;111: 107–123. https://doi.org.ezproxy.sussex.ac.uk/10.1037/0021-843X.111.1.107 11866165

[43] Browning M, Behrens TE, Jocham G, O’Reilly JX, Bishop SJ. Anxious individuals have difficulty learning the causal statistics of aversive environments. Nat Neurosci. 2015;18: 590–596. 10.1038/nn.3961 25730669 PMC4644067

[44] Huang H, Thompson W, Paulus MP. Computational Dysfunctions in Anxiety: Failure to Differentiate Signal From Noise. Biological Psychiatry. 2017;82: 440–446. 10.1016/j.biopsych.2017.07.007 28838468 PMC5576575

[45] Mkrtchian A, Aylward J, Dayan P, Roiser JP, Robinson OJ. Modeling Avoidance in Mood and Anxiety Disorders Using Reinforcement Learning. Biological Psychiatry. 2017;82: 532–539. 10.1016/j.biopsych.2017.01.017 28343697 PMC5598542

[46] Aylward J, Valton V, Ahn W-Y, Bond R, Dayan P, Roiser JP, et al Altered decision-making under uncertainty in unmedicated mood and anxiety disorders. 2018;PsyArXiv. 10.31234/osf.io/k5b8m PMC6790140 31209369

[47] Kim JJ, Fanselow MS. Modality-specific retrograde amnesia of fear. Science. 1992;256: 675–677. 10.1126/science.1585183 1585183

[48] Maren S, Aharonov G, Fanselow MS. Retrograde abolition of conditional fear after excitotoxic lesions in the basolateral amygdala of rats: Absence of a temporal gradient. Behavioral Neuroscience. 1996;110: 718–726. 10.1037//0735-7044.110.4.718 8864263

[49] de Berker AO, Rutledge RB, Mathys C, Marshall L, Cross GF, Dolan RJ, et al Computations of uncertainty mediate acute stress responses in humans. Nat Commun. 2016;7: 10996 10.1038/ncomms10996 27020312 PMC4820542

[50] Poulos AM, Li V, Sterlace SS, Tokushige F, Ponnusamy R, Fanselow MS. Persistence of fear memory across time requires the basolateral amygdala complex. PNAS. 2009;106: 11737–11741. 10.1073/pnas.0905257106 19567836 PMC2710655

[51] Lawson RP, Nord CL, Seymour B, Thomas DL, Dayan P, Pilling S, et al Disrupted habenula function in major depression. Molecular Psychiatry. 2017;22: 202–208. 10.1038/mp.2016.81 27240528 PMC5285459

[52] Lawson RP, Seymour B, Loh E, Lutti A, Dolan RJ, Dayan P, et al The habenula encodes negative motivational value associated with primary punishment in humans. PNAS. 2014; 201323586. 10.1073/pnas.1323586111 25071182 PMC4136587

[53] Lefebvre G, Lebreton M, Meyniel F, Bourgeois-Gironde S, Palminteri S. Behavioural and neural characterization of optimistic reinforcement learning. Nature Human Behaviour. 2017;1: 0067 10.1038/s41562-017-0067

[54] de Boer L, Axelsson J, Riklund K, Nyberg L, Dayan P, Bäckman L, et al Attenuation of dopamine-modulated prefrontal value signals underlies probabilistic reward learning deficits in old age. Schultz W, editor. eLife. 2017;6: e26424 10.7554/eLife.26424 28870286 PMC5593512

[55] Tzovara A, Korn CW, Bach DR. Human Pavlovian fear conditioning conforms to probabilistic learning. PLOS Computational Biology. 2018;14: e1006243 10.1371/journal.pcbi.1006243 30169519 PMC6118355

[56] Watanabe S. Asymptotic Equivalence of Bayes Cross Validation and Widely Applicable Information Criterion in Singular Learning Theory. J Mach Learn Res. 2010;11: 3571–3594.

[57] Betancourt MJ, Girolami M. Hamiltonian Monte Carlo for Hierarchical Models. arXiv:13120906 [stat]. 2013; Available: http://arxiv.org/abs/1312.0906

[58] Christakou A, Gershman SJ, Niv Y, Simmons A, Brammer M, Rubia K. Neural and Psychological Maturation of Decision-making in Adolescence and Young Adulthood. Journal of Cognitive Neuroscience. 2013;25: 1807–1823. 10.1162/jocn_a_00447 23859647

[59] Gottlieb J, Oudeyer P-Y, Lopes M, Baranes A. Information-seeking, curiosity, and attention: computational and neural mechanisms. Trends in Cognitive Sciences. 2013;17: 585–593. 10.1016/j.tics.2013.09.001 24126129 PMC4193662

[60] Renninger LW, Verghese P, Coughlan J. Where to look next? Eye movements reduce local uncertainty. Journal of Vision. 2007;7: 6–6. 10.1167/7.3.6 17461684

[61] Critchley HD, Mathias CJ, Dolan RJ. Neural Activity in the Human Brain Relating to Uncertainty and Arousal during Anticipation. Neuron. 2001;29: 537–545. 10.1016/s0896-6273(01)00225-2 11239442

[62] Diederen KMJ, Spencer T, Vestergaard MD, Fletcher PC, Schultz W. Adaptive Prediction Error Coding in the Human Midbrain and Striatum Facilitates Behavioral Adaptation and Learning Efficiency. Neuron. 2016;90: 1127–1138. 10.1016/j.neuron.2016.04.019 27181060 PMC4893165

[63] Symmonds M, Wright ND, Bach DR, Dolan RJ. Deconstructing risk: Separable encoding of variance and skewness in the brain. NeuroImage. 2011;58: 1139–1149. 10.1016/j.neuroimage.2011.06.087 21763444 PMC3176914

[64] Schultz W, Preuschoff K, Camerer C, Hsu M, Fiorillo CD, Tobler PN, et al Explicit neural signals reflecting reward uncertainty. Philosophical Transactions of the Royal Society of London B: Biological Sciences. 2008;363: 3801–3811. 10.1098/rstb.2008.0152 18829433 PMC2581779

[65] Hunt LT, Malalasekera WMN, Berker AO de, Miranda B, Farmer SF, Behrens TEJ, et al Triple dissociation of attention and decision computations across prefrontal cortex. Nature Neuroscience. 2018;21: 1471–1481. 10.1038/s41593-018-0239-5 30258238 PMC6331040

